# A Bioplex Analysis of Cytokines and Chemokines in First Trimester Maternal Plasma to Screen for Predictors of Miscarriage

**DOI:** 10.1371/journal.pone.0093320

**Published:** 2014-04-03

**Authors:** Natalie J. Hannan, Katerina Bambang, Tu’uhevaha J. Kaitu’u-Lino, Justin C. Konje, Stephen Tong

**Affiliations:** 1 Translational Obstetrics Group, The Department of Obstetrics and Gynaecology, Mercy Hospital for Women, University of Melbourne, Heidelberg, Victoria, Australia; 2 Endocannabinoid Research Group, Reproductive Sciences Section, Department of Cancer Studies and Molecular Medicine, University of Leicester, Leicester, United Kingdom; University of Leuven, Rega Institute, Belgium

## Abstract

**Background:**

We have previously shown in two independent cohorts that circulating first trimester Macrophage Inhibitory Cytokine-1 (MIC-1) levels are lower in women in early pregnancy who are destined to miscarriage. While promising, the diagnostic performance of measuring MIC-1 alone was not sufficient for it to be a useful predictive test for miscarriage. Besides MIC-1, there are other cytokines, as well as chemokines, involved in facilitating early pregnancy. We reasoned that screening these factors in maternal plasma could uncover other predictive markers of miscarriage.

**Methods:**

This was a nested case control study, of 78 women from a prospective study of 462 attending the Early Pregnancy Assessment Unit in the first trimester (EPAU) with a threatened miscarriage; 34 of these subsequently miscarried (cases) and 44 went on to have a normal delivery (controls) Cytokines IL-1β, IL-6 and IL-10, and the chemokines, CXCL8, CCL2, CCL5, CCL7 and CX3CL1 were measured in plasma from our cohort.

**Results:**

The cytokines IL-1β, IL-6, IL-10 and the chemokine CXCL8 were not detectable in first trimester plasma. The chemokines CCL2, CCL5, CCL7 and CX3CL1 were detectable in all samples but levels did not vary across 5–12 weeks of gestation among controls. Plasma levels of these chemokines were no different in the miscarriage cohort compared to controls.

**Conclusion:**

The chemokines CCL2, CCL5, CCL7 and CX3CL1 were detectable in plasma during the first trimester while IL-1β, IL-6, IL-10 and CXCL8 were not. However, none of the cytokines and chemokines screened were different in maternal plasma in cases or controls. These therefore do not appear to have potential for application as predictive biomarkers of miscarriage.

## Introduction

Miscarriage is the most common complication of pregnancy. There are currently no accurate predictive tests and treatments that can prevent spontaneous miscarriage. While 50% of miscarriages are associated with fetal chromosomal errors, most of the remaining cases are likely to be euploid fetuses that have failed owing to implantation problems [1]. We have previously postulated that developing an accurate predictive test for miscarriage may open the window for identifying euploid pregnancies that are still viable but destined to miscarry. It follows therefore that conceivably, emerging therapeutics could be targeted at such high risk euploid pregnancies so that some of them may continue to viability, (i.e. rescuing some from miscarriage). We [2]–[5], and others [6]–[8] have been avidly searching for such predictive blood biomarkers of miscarriage.

One such biomarker, macrophage Inhibitory cytokine 1 (MIC-1), is localized to the syncytiotrophoblast and decidua, increases across the first trimester in serum and is proposed to play an immunomodulatory role in facilitating pregnancy success. We have previously shown [2] and then validated in two large prospective cohorts 3,5 that MIC-1 is depressed in viable pregnancies destined for miscarriage. Given the success in the strategy of focusing on an immune cytokine with a likely role in early pregnancy, attempts need to be made to identify and screen other such molecules for their predictive potential.

Cytokines are small glycoprotein mediators which act locally, in both paracrine and autocrine manners. Chemokines are small chemotactic cytokines, well known for their function in leukocyte recruitment and activation. While cytokines and chemokines have major roles in regulating immune cells, these molecules are synthesized at the maternal-fetal interface where they have been implicated to play critical roles in the establishment and maintenance of pregnancy [9]–[13].

During the early stages of pregnancy it is thought that cytokines facilitate important immune modulation, resulting in a switch to the type 2 response, allowing the pregnancy to safely continue [14]. Disturbances in the production of individual cytokines have been demonstrated in the endometrium of some infertile women and in those suffering from recurrent miscarriage [15]–[19].

We therefore set out to screen cytokines and chemokines in maternal plasma, examining whether levels can predict miscarriage in pregnancies where the fetus was live on the day of blood sampling. The cytokines and chemokines (IL-1β, IL-6. IL-10, CCL2, CCL5, CCL7, CXCL8 and CX3CL1) chosen for examination in maternal blood in this study were selected because they have previously been reported to play key roles in the human endometrium [9], [10], [20]. In addition IL1 β, IL-6, IL-10 and CXCL8 (IL-8) have previously been examined in maternal circulation in association with miscarriage [21]–[23].

A subgroup of samples were selected from a larger prospective cohort of samples collected in the first trimester from women presenting to an Early Pregnancy Assessment Unit in the United Kingdom. Notably, all participants were confirmed to have a viable pregnancy by ultrasound on the day of blood sampling. We assessed eight cytokines and chemokines, to examine (1) whether they were detectable and to document their levels across the first trimester and (2) whether these were different in women destined to later miscarry and thus maybe used as biomarkers of spontaneous miscarriage.

## Materials and Methods

### Participants

This was a nested case control study, in which we selected samples from well phenotyped women from those obtained for a large prospective cohort study. The original prospective collection was performed at the Leicester Royal Infirmary, were of women presenting to the Early Pregnancy Assessment Unit (EPAU) either most commonly with a threatened miscarriage or a past history of previous miscarriage or ectopic pregnancy. Women presented with pain and/or bleeding or were asymptomatic. Women were excluded if they had a history of illicit dug use, any significant systemic illness such as diabetes or if they were heavy smokers (>20 cigarettes per day). Only singleton pregnancies were included. Gestational age was determined by ultrasound measurement of the crown-rump length and calculating the gestation using reference British Medical Ultrasound Society (BMUS) charts, all patients were recruited and assessed by a single operator. Fetal cardiac activity was confirmed at the time of blood sampling. A miscarriage was defined as the spontaneous loss of the pregnancy at less than 20 weeks gestation, a first trimester miscarriage is the spontaneous loss of the pregnancy less than 13 weeks gestation.

The blood analysed was collected at 5–12 weeks gestation (during the first trimester). In addition to the blood samples, various demographic variables were also collected as detailed in Table 1.

**Table 1:** Characteristics of study participants.

	Controls (n = 44)	Miscarriage (n = 34)
**Maternal age (years)**	31 (21–43)	33 (20–42)
**Body Mass Index**	25 (20–40)	26 (18–41)
**Ethnicity**		
***Caucasian***	33 (75%)	28 (63.6%)
***Caucasian other***	3 (6.8%)	
***Asian***	5 (11.4%)	3 (8.8%)
***Black***	1 (2.7%)	
***Mixed***	2 (4.5%)	
***Not recorded***		3 (8.8%)
**Parity %**		
**0**	52	35
**1**	33	33
**≥2**	16	32
**Smoker**	3 (6.8%)	6 (17.6%)
**Gestation at sampling (wks+days)**	7 (5+0–12+0)	6+6 (5+0–12+0)
**Previous miscarriages**	2 (0–6)	1 (0–4)
**Gestation at delivery (wks+days)**	39.6+3 (32+0–42+0)	n/a
**Average birth weight (g)**	3350 g (1900–4340)	n/a

Data provided as the median, with range given in brackets or percentage as indicated.

The original a priori study design was to perform a bioplex screen of eight candidate cytokines and chemokines and then validate the predictiveness of spontaneous miscarriage using promising leads (i.e. cytokines or chemokines with differential levels among the two cohorts) in the entire cohort of 462.

### Ethics Statement

This study was approved by the Leicestershire and Rutland local research ethics committee. All women gave informed written consent to participate in the study.

### Sample Collection

Approximately 9 mL of venous blood was collected into EDTA plasma tubes (Sarstedt Leicester UK). The tubes were centrifuged at 1200×*g* at 4°C for 30 minutes, and the plasma collected and stored in aliquots at –80°C until analysis. All samples were promptly processed and frozen within 60 minutes of collection.

### Measurement of Cytokines IL-1β, IL-6, IL-10; the Chemokines CCL2, CCL5, CCL7, CXCL8 and CX3CL1; and Human Chorionic Gonadotrophin (hCG)

The cytokines, IL-1β, IL-6 and IL-10, and the chemokines, CXCL8, CCL2, CCL5, CCL7 and CX3CL1 were measured using quantitative Milliplex Luminex (MilliPlex MAP Human Cytokine Panel 8-plex, Millipore, Melbourne, Victoria, Australia) assays according to the manufacturer’s instructions. A single operator blinded to the clinical groupings performed all analyte measurements. In brief, 96-well plates were pre-wet with 200 μl assay buffer (provided by the manufacturer) for 10 minutes and then aspirated using a vacuum manifold. Standards and plasma samples (25 μl) were added to appropriate wells, followed by the addition of assay beads. Plates were incubated overnight for 16–18 h with mild agitation at 4°C; the fluid was then removed by vacuum and the wells were washed twice with wash buffer. Detection antibodies were added to each well, and incubated for 1 h at room temperature (RT), the fluorescent conjugate Streptavidin-Phycoerythrin was added to each well and plates incubated for 30 min at RT. Fluid was then removed by vacuum and wells washed twice. Analysis of each sample was performed in duplicate. Identical positive and negative quality controls are included on each assay in duplicate.

The assays were performed in one batch, with samples randomly mixed. The lower detection limit was 3.2 pg/ml for all the analytes, while the intra-assay variability was less than 10%. Data were collected and analysed using a BioPlex 200 instrument equipped with BioManager analysis software (BioRad).

Levels of hCG were also assayed using ELISA (ALPCO, Salem, NH, USA).

### Statistical Analysis

Data were not normally distributed and were therefore assessed non-parametrically. Mann-Whitney U test was used to assess the demographic characteristics for maternal age, BMI, Smoker, gestation at sampling, previous miscarriages. Chi-squared contingency analysis was used to assess the demographic characteristics for Ethnicity and Parity. Plasma cytokine and chemokine concentrations were assessed across gestation using the Kruskal-Wallis test. The Mann-Whitney U test was used to compare plasma cytokine and chemokine concentrations between the miscarriage cohort and controls.

The analyte concentrations for hCG varied across gestation. To correct for this change across gestational age, we also expressed all data points for hCG as multiple of the normal median (MoMs). We then compared MoMs between miscarriage and controls using the Mann-Whitney U test. All statistical analysis was carried out using PRISM version 6.00 for Mac (GraphPad, SanDiego, CA). p<0.05 was considered significant.

## Results

### Patient Characteristics

We examined a set of cases and controls of 78 samples selected from a large prospective collection. This comprised 44 controls (women who progressed to a live baby at term) and 34 miscarriage samples. All miscarriages were diagnosed in the first trimester. Table 1 shows the baseline demographics for the cases and controls. The median maternal age (and range) for the control group was 31 years, and was no different to the miscarriage cohort. There were also no significant differences in parity, or the number of previous miscarriages. All women in the ongoing pregnancy cohort progressed to delivery of a live born infant, with most delivering at term.

Samples were grouped according to gestational age at the time of blood collection. For control group, there were: n = 6 at 5 weeks; n = 10 at 6 weeks, n = 6 at 7 weeks; n = 5 at 8 weeks; n = 5 at 9 weeks; n = 4 at 10 weeks; n = 4 at 11 weeks; n = 4 at 12 weeks. The gestational ages of the miscarriage group (cases) were 5–12 weeks with n = 3 at 5 weeks; n = 20 at 6 weeks; n = 3 at 7 weeks; n = 6 at 8 weeks; n = 1 at 9 weeks and n = 1 at 12 weeks.

### Maternal Plasma Cytokine and Chemokine Levels at 5–12 Weeks Gestation in Controls

IL-1β, IL-6, IL-10, and CXCL8 were all largely undetectable in maternal plasma (below the limit of detection of the assay for the majority of samples). These analytes were therefore omitted from further analysis.

**Figure 1:**
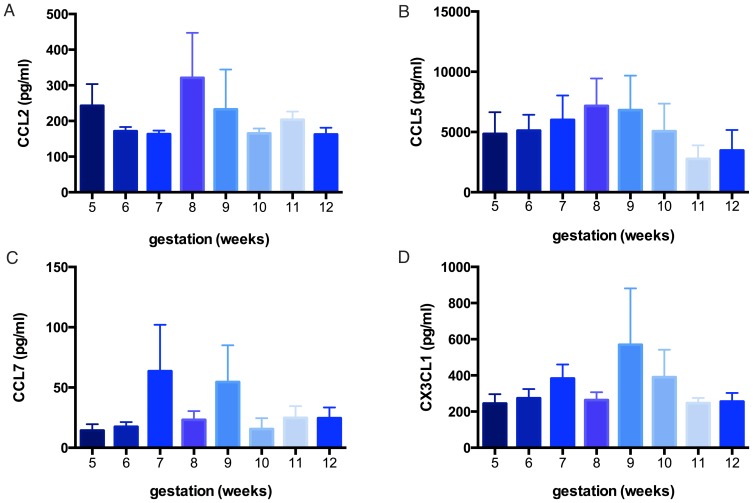
Analysis of plasma CCL2, 5 and 7 and CX3CL1 (A, B, C and D respectively) across weeks 5–12 in the control cohort (ongoing pregnancies). None of the chemokines varied significantly across the gestational weeks (N.S. by Kruskal-Wallis test, all analyses P≥0.39). Data expressed as mean ± SEM.

The chemokines CCL2, 5 and 7 and CX3CL1 were detectable in the maternal plasma, but did not alter significantly across during the study period i.e.5 to 12 weeks’ gestation (Figure 1A–D) The mean (±SEM) maternal plasma CCL2 concentrations in the control group were 207 (±21) pg/ml. The mean (±SEM) CCL5 plasma levels in the control group were 5258 (±671) pg/ml (Figure 1B) while the mean CCL7 plasma levels were 29 (±7) pg/ml (Figure 1C), and mean CX3CL1 levels were 324 (±42) pg/ml.

### Maternal Plasma Levels of the Chemokines, CCL2, 5 and 7 and CX3CL1 at 5–12 Weeks Gestation in the Cases

**Figure 2:**
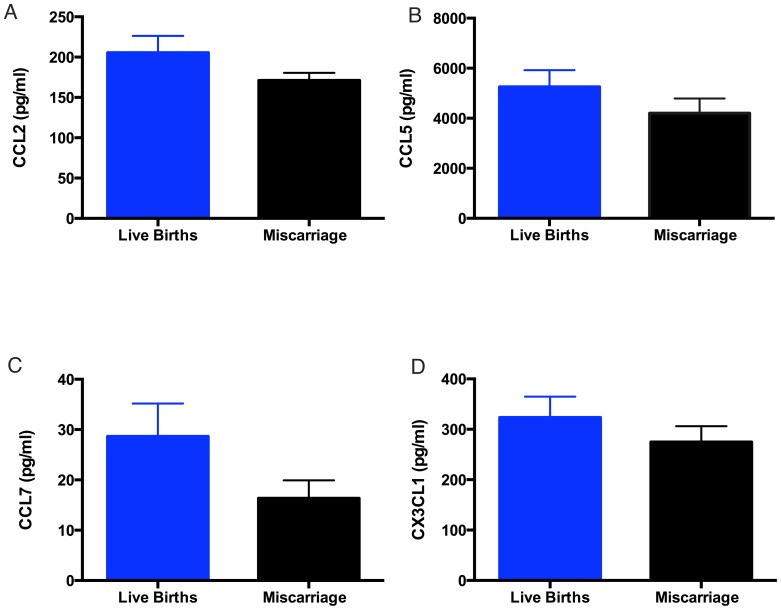
Analysis of plasma CCL2, 5 and 7 and CX3CL1 (A, B, C and D respectively) in normal control pregnancy (white bars) and miscarriage (black bars) cohorts. Chemokine levels did not significantly differ between normal control pregnancies and the miscarriage cohort. (N.S. by Kruskal-Wallis test, all analyses P≥0.14). Data expressed as mean ± SEM.

There were no significant differences in maternal plasma chemokine CCL2, 5, 7 and CX3CL1 in the cases (women with a viable pregnancy at blood sampling but who subsequently miscarried) compared to that in women who delivered a live baby at term (Figure 2).

### Plasma hCG Concentrations

Since there were no significant differences in the 8 analytes screened, to ensure that our samples were representative of the proteome seen in miscarriage, we measured hCG as we expected the levels to be lower in the miscarriage cohort relative to controls. The hCG levels reported here were obtained from a larger cohort of samples which have been already published (this specific subcohort hCG analysis has not been reported previously).

**Figure 3:**
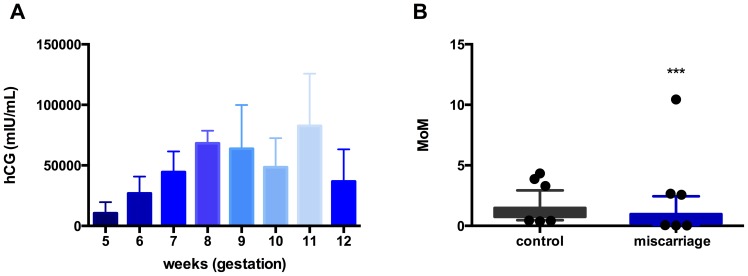
Plasma human chorionic gonadotrophin concentrations in controls (white bars) and miscarriage cohort. Figure 3A shows plasma hCG concentrations across gestation in the control cohort (P = 0.0011, Kruskal-Wallis test). 3B shows a comparison of plasma hCG levels in the miscarriage and control cohorts, expressed as multiples of the median (MoMs). Use of MoMs corrects for the significant variations in analyte concentrations across gestation. ***P<0.0001. The hCG levels reported here were obtained from a larger cohort of samples which have been already published [5] (this specific subcohort hCG analysis has not been reported previously).

Plasma hCG levels in the control cohort peaked at 8 weeks gestation and started then falling (Figure 3A). To correct for changes across gestation, the data were expressed as multiples of the median (of the controls) [3]. This confirmed that plasma hCG levels in the miscarriage cohort were significantly lower compared to that in the control group (Figure 3B), at 35% of normal levels compared to controls (Median and 25–75^th^ centile) MoM in the miscarriage cohort was 0.35 (0.21–0.97) vs 1.00 (75–1.48) in controls; p<0.0001.

## Discussion

Miscarriage is the most common complication of pregnancy, affecting around 10–15% of clinical pregnancies. While there is no therapeutic treatment to prevent most miscarriages, there continues to be keen interest in identifying one [24], [25]. The development of a non-invasive test to identify women at high risk of miscarriage at an early stage of pregnancy, before symptoms occur, would be valuable. Such a test could allow targeted evaluation of potential therapies, limiting the exposure to women at high risk for miscarriage. This would be particularly important when potential treatments would need to be administered during the time of embryogenesis. A further clinical application of a predictive test would be to use it as a counseling tool when, potentially, anxious women may be reassured or, alternately, warned they are at risk of a spontaneous miscarriage at a very early stage of pregnancy. It is in this context that our team has had a continuing interest in searching for predictive biomarkers for miscarriage [2]–[5].

Cytokines and chemokines have been shown to play key roles during early implantation events [26]–[30] and their receptors are abundantly expressed by human trophoblast [11]–[13], [31]. Previously we and others have demonstrated that CCL2, 5, and 7 and CX3CL1 are produced by a variety of cell types in the maternal endometrium and have key local actions important for implantation including leukocyte infiltration and trophoblast trafficking [9], [10], [12], [20], [32]. We demonstrated that CX3CL1 promotes migration of human trophoblast *in vitro* and neutralizing CX3CL1 attenuated trophoblast migration in response to endometrial cell conditioned media [11]. Furthermore, invasive trophoblasts have been shown to acquire the chemokine receptor CCR1 as they invade the maternal wall, providing their ligands (including CCL5) a binding site and enhancing trophoblast migration during placentation [33]. Importantly all chemokines examined in maternal blood in this study have previously been reported to be present in the human endometrium 9,10,20.

MIC-1 has been found to be highly expressed in the placenta and increases during pregnancy. Furthermore, functional studies have suggested that MIC-1 promotes a tolerogenic subtype of dendritic cells in the decidua [34]. We have previously shown in three independent cohorts that MIC-1 is depressed in association with miscarriage [2], [3], [5]. Furthermore, it appears to have a reasonable diagnostic performance characteristics for miscarriage prediction when applied to an asymptomatic cohort. Thus, these studies of MIC-1 represent an important proof of concept: measuring an analyte known to be likely involved in the biology of pregnancy has lead to the identification of a blood marker differentially produced in association with miscarriage. It is for this reason that we hypothesized that screening other cytokines and chemokines might yield further potential biomarkers of miscarriage.

Using a prospective collection of maternal plasma samples, we assessed levels of pro-inflammatory cytokines IL-1β, IL-6. IL-10 and the chemokines CCL2, CCL5, CCL7, CXCL8 and CX3CL1 in women (both symptomatic and asymptomatic) with viable pregnancies who went on to miscarry. Unfortunately, we found none of these analytes to be promising markers of miscarriage. Most of the samples assessed had undetectable levels of IL-1β, IL-6, IL-10 and CXCL8. Previously levels of IL-6, CXCL8 and IL-10 have been detected in maternal blood [21], [23], [35], [36]. Key parameters differ between these studies and the current study; many looked at maternal serum [21], [35] where as the current study examines plasma levels, this may account for the fact we cannot detect these cytokines in the current study. Other reports have noted low and non-detectable circulating levels of several cytokines including IL-1β, IL-6, IFN-γ and TNF-α and lack of consistent associations with miscarriage risk [37], [38]. A further plausible explanation for inconsistency with this study and others may be related to timing of sample collection relative to miscarriage and also the time taken to process the sample. In our study all plasma samples were collected when the patient was still pregnant (presence of fetal heart beat at ultrasound) and all samples were processed, and frozen (in aliquots) within 1 hour of collection.

The chemokines CCL2, CCL5, CCL7 and CX3CL1 were readily detectable in the maternal circulation, but levels were not different in controls and cases. We note that our findings do not preclude the possibility they are still likely to play important biological roles in the local uterine environment, where they are produced in abundance by various maternal endometrial cells in both non-pregnant and pregnant tissue [9]–[13], [17], [33], [39].

Our study has a number of strengths; the samples were chosen from a larger prospective study where the specific goal was to identify biomarkers of miscarriage. Importantly to assess gestational age, at the time of blood sampling, all ultrasounds were performed by the same investigator. This approach eliminated potential error arising from inter-observer variability caused by different people performing the ultrasounds to determine gestational age (a potential weakness present in other studies). All samples were collected and processed by the same investigator for storage. Furthermore, the analytes were measured as a batch in a single run by an operator who mixed the samples and was blinded to the clinical groupings. Finally, we had hCG levels in these samples which showed an expected lower level in the miscarriage cohort. This provides reassurance that our selected samples were probably representative of the miscarriage phenotype seen in the clinic.

While the cytokines screened in the current study may not be useful to predict spontaneous pregnancy loss, our data does not preclude their possible clinical utility in prediction of future pregnancy loss in those who suffer from recurrent miscarriage, particularly where the pathogenic mechanism may be very different.

In conclusion, we have found plasma concentrations of the cytokines IL-1β, IL-6 and IL-10, and the chemokines, CXCL8, CCL2, CCL5, CCL7, CX3CL1 cannot predict subsequent miscarriage.
